# Association of plasma chromium with metabolic syndrome among Chinese adults: a case-control study

**DOI:** 10.1186/s12937-020-00625-w

**Published:** 2020-09-23

**Authors:** Sijing Chen, Li Zhou, Qianqian Guo, Can Fang, Mengke Wang, Xiaobo Peng, Jiawei Yin, Shuzhen Li, Yalun Zhu, Wei Yang, Yan Zhang, Zhilei Shan, Xiaoyi Chen, Liegang Liu

**Affiliations:** 1 Department of Nutrition and Food Hygiene, Hubei Key Laboratory of Food Nutrition and Safety, School of Public Health, Tongji Medical College, Huazhong University of Science and Technology, Wuhan, 430030 Hubei China; 2 Ministry of Education Key Lab of Environment and Health, School of Public Health, Tongji Medical College, Huazhong University of Science and Technology, Wuhan, 430030 Hubei China; 3 The Hubei Provincial Key Laboratory of Yeast Function, Yichang, 443003 Hubei China; 4 Department of Nutrition and Food Hygiene, School of Public Health, Guangzhou Medical University, Guangzhou, 511436 China

**Keywords:** Chromium, Metabolic syndrome, High blood glucose, Lipid

## Abstract

**Backgroud:**

Chromium has been suggested playing a role in alleviating diabetes, insulin resistance and lipid anomalies, but the effect on metabolic syndrome (MetS) in humans remains controversial.

**Methods:**

We conducted a matched case-control study in a Chinese population, involving 2141 MetS cases and 2141 healthy controls, which were 1:1 matched by age (±2 years) and sex. Plasma chromium was measured by inductively coupled plasma mass spectrometry.

**Results:**

Plasma chromium levels were lower in MetS group than in control group (mean: 4.36 μg/L and 4.66 μg/L, respectively, *P* < 0.001), and progressively decreased with the number of MetS components (*P* for trend < 0.001). After adjustment for potential confounding factors, the odds ratios (95% confidence intervals) for MetS across increasing quartiles of plasma chromium levels were 1 (reference), 0.84 (0.67–1.05), 0.76 (0.61–0.95), and 0.62 (0.49–0.78), respectively (*P* for trend < 0.001). For the components of MetS (high waist circumference, high triglycerides and high blood glucose), the odds ratios (95% confidence intervals) of the highest quartiles were 0.77 (0.61–0.95), 0.67 (0.55–0.80), and 0.53 (0.44–0.64), respectively (*P* for trend < 0.05).

**Conclusions:**

Our results indicated that plasma chromium levels were inversely associated with MetS in Chinese adults. The association may be explained by the relations between plasma chromium levels and high waist circumference, and the triglycerides and blood glucose levels.

## Introduction

Metabolic syndrome (MetS), known as a constellation of metabolic abnormalities, which includes abdominal obesity, high triglycerides, low high-density lipoprotein (HDL) cholesterol, high blood pressure, and elevated fasting blood glucose, is now both a public health and a clinical problem. MetS is epidemic all over the world and its incidence has been rising year-on-year [1]. Recent data indicated that about 33.9% of the adults in Mainland China had MetS [2]. In addition, MetS has been realized a major contributor to the epidemic of cardiovascular disease and type 2 diabetes mellitus [3], and it may increase the risk of mortality [4].

Chromium is an essential trace element, which has been suggested playing a potential role in alleviating diabetes, insulin resistance and lipid anomalies. The beneficial mechanism has been investigated in experimental studies [5–9]. However, the epidemiological evidence of the protective effect of chromium on MetS is very limited, and has inconsistent conclusion so far. A prospective study suggested an inverse association between chromium and incidence of MetS in American young adults, and the inverse association was mainly explained by its relation to blood lipids [10]. There was another case-control study suggesting an association between low chromium levels and increased risk of nonfatal myocardial infarction [11]. Besides, our previous study found that plasma chromium concentrations were inversely associated with type 2 diabetes mellitus and pre-diabetes mellitus [12]. Yet some studies did not support the inverse relationship between chromium and MetS [13, 14]. So far, clinical trials evaluating chromium supplementation on glucose and lipid profiles have yielded conflicting results [15–18].

Accordingly, in this matched case-control study, we aimed to examine the association of plasma chromium levels with MetS along with its components in a large Chinese population.

## Methods

### Study population

The present study was a case-control study conducted in Wuhan, China, during the period of March 2013 to December 2017. The study population consisted of 2141 MetS cases and 2141 healthy controls, which were 1:1 matched by age (±2 years) and sex. All participants were aged 18 years or older, consecutively recruited from the general population undergoing a routine health checkup in the Tongji Hospital of Tongji Medical College, Huazhong University of Science and Technology. Patients with clinical significant neurological, endocrinological or other systemic diseases, as well as acute illness and chronic inflammatory or infective diseases were excluded from the study. All the participants enrolled were of Chinese Han ethnicity. All subjects gave their informed consent for inclusion before they participated in the study. The study was conducted in accordance with the Declaration of Helsinki, and the protocol was approved by the Ethics Committee of Tongji Medical College.

### Definition of MetS

The definition of MetS was based on the harmonized definition for MetS in 2009 [19]. To be considered as a case of MetS, participants had to meet at least three of the following criteria: 1. Abdominal obesity: waist circumference ≥ 85 cm in men and ≥ 80 cm in women; 2. Hypertriglyceridemia: ≥ 150 mg/dL; 3. Low levels of HDL cholesterol: < 40 mg/dL in men and < 50 mg/dL in women; 4. High blood pressure: ≥ 130/85 mmHg and/or use of antihypertensive medication; 5. High fasting glucose: ≥ 100 mg/dL and/or current use of antidiabetic medication and/or self-reported history of diabetes. The controls had zero to two components of MetS which were mentioned above.

### Data collection

Demographics, health status, and lifestyle data were obtained from the questionnaires, including sex, age, education level, history of disease (diabetes, hypertension and hyperlipemia), family history of diabetes, physical activity, current smoking status, and current alcohol drinking status. Education level was classified as none or elementary school, middle school, and high school or college. Physical activity was classified as at least once/week or no. Current smoking status was classified as yes (at least one cigarette per day over the previous 6 months) or no. Current alcohol drinking status was classified as yes (drink alcohol beverage more than once a week over the previous 6 months) or no. Anthropometric data including height (m), mass (kg), waist circumference and blood pressure were measured with standardized techniques by trained and certified technicians. BMI (body mass index) was calculated as mass divided by the square of height (kg/m^2^). Waist circumference was obtained at the mid-point between the lowest rib and the iliac crest to the nearest 0.1 cm, after inhalation and exhalation. Hip circumference was measured at the outermost points of the greater trochanters. The ratio of waist-to-hip circumference was used as an index of fat distribution. Blood pressure was measured at rest in the seated position using a standardized automated sphygmomanometer after 5 min of rest, and repeated in both arms.

### Laboratory measurements

Blood samples were collected in all participants after an overnight fast of at least 10 h. Details of measurement of fasting plasma glucose, fasting plasma insulin, total cholesterol, triglyceride, HDL cholesterol, low-density lipoprotein (LDL) cholesterol and calculation of homeostasis model assessment of insulin resistance (HOMA-IR) and HOMA of β-cell function (HOMA-β) have been described previously [20]. Plasma malonaldehyde (MDA) was measured with an MDA assay kit (Jiancheng, Inc., Nanjing, China).

### Measurement of plasma chromium concentrations

Plasma chromium concentrations were measured in the Ministry of Education Key Laboratory of Environment and Health and School of Public Health at Tongji Medical College of Huazhong University of Science and Technology, using inductively coupled plasma mass spectrometry (ICP-MS) (Agilent 7700 Series, Tokyo, Japan). Plasma samples were stored at − 80 °C. The case and control specimens were measured randomly in the daily measurement, with laboratory personnel blinded to the case–control status. For quality assurance, metals in standard reference materials were measured once in every 20 samples using certified reference material. The certified concentrations of human plasma controls (ClinChek no. 8883 and 8884) were 3.56 ± 0.89 μg/L and 11.1 ± 2.22 μg/L, respectively. The limit of detection (LOD) for chromium was 0.01 μg/L, and concentrations of plasma chromium levels below the LOD (0.7%) were imputed at LOD/√2. Quality control was performed (1 out of 20 samples), and the inter-assay and intra-assay coefficients of variation were < 10 and < 8%, respectively.

### Statistical analysis

Descriptive statistics were calculated for all demographic and clinical characteristics of the study subjects, and summarized as numbers (percentages) for categorical data, mean ± standard deviations (SDs) for normally distributed data, and medians (interquartile ranges) for non-normally distributed data. Comparisons between MetS and controls were performed by *t* test or Mann-Whitney *U* test for continuous variables, and chi-square tests for categorical variables. In addition, subjects were divided into 6 groups according to their possession of 0, 1, 2, 3, 4 or 5 components of MetS. Multiple imputation based on 5 replications and a fully conditional specification method in SPSS was used to account for missing data.

For calculation of the odds ratio (OR) for MetS, plasma chromium concentration was categorized in quartiles according to the control group: category 1, < 3.27 μg/L; category 2, 3.28–4.46 μg/L; category 3, 4.47–5.87 μg/L, and category 4, > 5.88 μg/L. Conditional logistic regression was used to assess the association of MetS with plasma chromium concentrations. The ORs and 95% confidence intervals (CIs) of MetS were calculated between the quartiles of chromium using the lowest quartile as the reference category, and also by per 1 μg/L chromium as continuous variable. We considered three models with progressive degrees of adjustment: model 1 adjusted for age; model 2 additionally adjusted for education, current smoking status, current alcohol drinking status, physical activity and family history of diabetes; and model 3 further adjusted for BMI. Tests of linear trend across increasing chromium quartiles were conducted by assigning the median value to each quartile and treating it as a continuous variable. Furthermore, the ORs of the MetS components including high waist circumference, high triglycerides, low HDL cholesterol, high blood pressure, and high blood glucose were calculated using binary logistic regression.

To evaluate the consistency of the association between chromium and MetS by participant characteristics, additional analyses were run, stratifying age (< 50, ≥50), sex, BMI (< 24, ≥24), physical activity, current smoking status, and current drinking alcohol status. The interactions between these stratification variables and plasma chromium were tested by adding multiplicative terms into the multivariate logistic regression models; the likelihood ratio tests were conducted to examine the interactions.

Statistical analyses were performed with SPSS for Windows, version 24.0 (SPSS Inc., Chicago, Illinois). *P* values reported are two tailed, and values below 0.05 were considered statistically significant.

## Results

Anthropometric and metabolic characteristics of the 2141 MetS and 2141 controls are reported in Table 1. Compared with control subjects, the individuals with MetS had higher prevalence of family history of diabetes and lower rate of smoking and activity (*P* < 0.05). As expected, we observed higher levels of BMI, waist circumference, hip circumference, waist-to-hip circumference ratio, systolic blood pressure (SBP), diastolic blood pressure (DBP), fasting plasma glucose, fasting plasma insulin, HOMA-IR, triglycerides, total cholesterol, LDL cholesterol and lower levels of HDL cholesterol in MetS than in the controls (*P* < 0.001). MetS group had higher MDA levels than the control group (*P* < 0.001).

**Table 1:** Anthropometric and metabolic characteristics of controls and MetS

Parameters	MetS (*n* = 2141)	controls (*n* = 2141)	*P* value
Male, n (%)	1293 (60.4)	1293 (60.4)	1.000
Age (y)	52.57 ± 10.80	52.70 ± 10.73	0.750
BMI (kg/m^2^) ^a^	25.82 ± 3.10	22.66 ± 2.62	< 0.001
Waist circumference (cm)	89.46 ± 8.67	79.98 ± 7.90	< 0.001
Hip circumference (cm)	98.49 ± 6.32	92.78 ± 6.04	< 0.001
Waist / hip ratio	0.91 ± 0.05	0.86 ± 0.06	< 0.001
SBP (mmHg)	140.14 ± 20.36	128.80 ± 19.11	< 0.001
DBP (mmHg)	84.19 ± 12.06	77.12 ± 10.42	< 0.001
Current smoker, n (%) ^b^	617 (28.8)	700 (32.7)	0.006
Current drinker, n (%) ^c^	604 (28.4)	621 (29.2)	0.578
Physical activity, n (%) ^d^	746 (38.1)	884 (42.2)	0.006
Family history of diabetes, n (%) ^e^	309 (14.6)	158 (7.5)	< 0.001
Educational level, n (%) ^f^			0.308
None or elementary school	402 (20.6)	426 (20.3)	
Middle school	791 (40.6)	901 (42.9)	
High school or college	754 (38.7)	772 (36.8)	
Fasting plasma glucose (mmol/L)	7.12 ± 2.57	5.66 ± 1.33	< 0.001
Fasting plasma insulin (mmol/L)	10.66 (7.58–15.40)	6.87 (4.79–9.95)	< 0.001
HOMA-IR	3.34 (2.20–5.04)	1.72 (1.20–2.53)	< 0.001
HOMA-β	69.09 (42.00–106.44)	67.36 (46.81–98.60)	0.688
Triglycerides (mmol/L)	4.89 ± 1.15	4.71 ± 0.87	< 0.001
Total cholesterol (mmol/L)	1.96 (1.39–3.05)	1.14 (0.84–1.49)	< 0.001
HDL cholesterol (mmol/L)	1.12 (0.91–1.32)	1.41 (1.25–1.61)	< 0.001
LDL cholesterol (mmol/L)	2.72 ± 1.14	2.46 ± 0.88	< 0.001
MDA (nmol/L)	4.82 (3.95–5.98)	4.32 (3.62–5.27)	< 0.001

Data are shown in numbers (percentages), mean ± standard deviations, or medians (interquartile ranges). *P* values were obtained from *t* test, Mann-Whitney *U* test, or chi-square test, where appropriate

All biomarkers detected were fasting

^a^ Missing number was 28. ^b^ Missing number was 5. ^c^ Missing number was 28. ^d^ Missing number was 238. ^e^ Missing number was 50. ^f^ Missing number was 236

Abbreviations: *MetS* Metabolic syndrome, *BMI* Body mass index, *SBP* Systolic blood pressure, *DBP* Diastolic blood pressure, *HOMA-IR* Homeostasis model assessment of insulin resistance, *homa-β* Homeostasis model assessment of β-cell function, *HDL* High-density lipoprotein, *LDL* Low-density lipoprotein, *MDA* Malonaldehyde

Plasma chromium concentrations were significantly decreased in the individuals with MetS compared with controls (mean: 4.36 μg/L in MetS, and 4.66 μg/L in controls, *P* < 0.001). For the 5 components of MetS, participants with high triglycerides and high blood glucose had significant lower levels of plasma chromium (*P* < 0.001). Furthermore, plasma chromium levels progressively decreased with the number of MetS components (*P* for trend < 0.001) (Table 2).

**Table 2:** Plasma chromium levels according to presence or absence and the number of MetS components

	Chromium (μg/L)		*P* value
	Present	Absent	
Metabolic syndrome	4.36 ± 2.37	4.66 ± 2.37	< 0.001
High waist circumference ^a^	4.45 ± 2.18	4.57 ± 2.30	0.081
High triglycerides ^b^	4.31 ± 2.15	4.63 ± 2.29	< 0.001
Low HDL cholesterol ^c^	4.45 ± 2.06	4.53 ± 2.33	0.263
High blood pressure ^d^	4.51 ± 2.29	4.39 ± 2.26	0.120
High blood glucose ^e^	4.34 ± 2.16	4.80 ± 2.37	< 0.001
No. of MetS components			
0	4.84 ± 2.30		
1	4.69 ± 2.26		
2	4.56 ± 2.48		
3	4.43 ± 2.19		
4	4.23 ± 1.92		
5	4.44 ± 2.22		
*P* for trend	< 0.001		

Data are shown in mean ± standard deviations. *P* values were obtained from *t* test. *P* for trend was obtained by one-way ANOVA

All biomarkers detected were fasting

^a^ Waist circumference ≥ 85 cm in men and ≥ 80 cm in women. ^b^ Triglyceridemia ≥150 mg/dL. ^c^ HDL cholesterol < 40 mg/dL in men and < 50 mg/dL in women. ^d^ Blood pressure ≥ 130/85 mmHg and/or use of antihypertensive medication. ^e^ Fasting blood glucose ≥100 mg/dL and/or current use of antidiabetic medication and/or self-reported history of diabetes

Abbreviations: *MetS* Metabolic syndrome, *HDL* High-density lipoprotein

Significant inverse associations between the levels of plasma chromium concentration and MetS were observed, and multiple adjusted models showed similar results (Table 3). After overall multivariable adjustment of age, education, current smoking status, current alcohol drinking status, physical activity, family history of diabetes, and BMI, the ORs (95% CIs) for MetS from the lowest to the highest quartiles were 1 (reference), 0.84 (0.67–1.05), 0.76 (0.61–0.95), and 0.62 (0.49–0.78), respectively (*P* for trend < 0.001). When plasma chromium concentration was considered as a continuous variable, the overall OR (95% CI) of having MetS was 0.95 (0.92–0.98) per 1 μg/L increment of chromium concentration.

**Table 3:** Association of plasma chromium concentrations with MetS

Variables	Quartiles of plasma chromium concentrations				Per 1 μg/L Chromium	*P* for trend
	Q1	Q2	Q3	Q4		
Chromium (μg/L)	< 3.27	3.28–4.46	4.47–5.87	> 5.88		
Cases/Controls, n/n	664 / 535	533 / 535	530 / 536	414 / 535		
Model 1	1	0.80 (0.68–0.95)	0.80 (0.67–0.94)	0.61 (0.52–0.73)	0.95 (0.93–0.98)	< 0.001
Model 2	1	0.80 (0.67–0.95)	0.80 (0.67–0.94)	0.62 (0.52–0.74)	0.95 (0.93–0.98)	< 0.001
Model 3	1	0.84 (0.67–1.05)	0.76 (0.61–0.95)	0.62 (0.49–0.78)	0.95 (0.92–0.98)	< 0.001

Model 1: adjusted for age;

Model 2: additionally adjusted for education, current smoking status, current alcohol drinking status, physical activity and family history of diabetes;

Model 3: additionally adjusted for BMI

Abbreviations: *MetS* Metabolic syndrome, *SD* Standard deviation

The associations of plasma chromium concentrations with each component of MetS were examined afterwards. Similar inverse associations were observed in high waist circumference, high triglycerides and high blood glucose, and the full adjusted ORs (95% CIs) of the highest quartiles were 0.77 (0.61–0.95), 0.67 (0.55–0.80), and 0.53 (0.44–0.64), respectively (*P* for trend < 0.05) (Table 4). As for low HDL cholesterol, significant associations were observed in model 1, but not in model 2 and 3. Association of plasma chromium concentrations with high blood pressure was not found in this study (Table 4).

**Table 4:** Association of plasma chromium concentrations with components of MetS

Variables	Quartiles of plasma chromium concentrations				Per 1 μg/L Chromium	*P* for trend
	Q1	Q2	Q3	Q4		
Chromium (μg/L)	< 3.27	3.28–4.46	4.47–5.87	> 5.88		
High waist circumference						
Model 1	1	0.83 (0.70–0.98)	0.92 (0.78–1.09)	0.77 (0.64–0.91)	0.98 (0.96–1.00)	0.013
Model 2	1	0.84 (0.71–1.00)	0.94 (0.79–1.11)	0.78 (0.66–0.93)	0.98 (0.96–1.00)	0.004
Model 3	1	0.87 (0.70–1.08)	0.88 (0.71–1.09)	0.77 (0.61–0.95)	0.99 (0.95–1.01)	0.035
High triglycerides						
Model 1	1	0.73 (0.62–0.87)	0.68 (0.57–0.81)	0.67 (0.56–0.80)	0.97 (0.94–0.99)	< 0.001
Model 2	1	0.72 (0.60–0.85)	0.67 (0.56–0.79)	0.66 (0.55–0.79)	0.96 (0.94–0.99)	< 0.001
Model 3	1	0.72 (0.60–0.86)	0.64 (0.54–0.77)	0.67 (0.55–0.80)	0.97 (0.94–0.99)	< 0.001
Low HDL cholesterol						
Model 1	1	1.09 (0.91–1.30)	1.13 (0.95–1.35)	0.80 (0.66–0.96)	0.99 (0.97–1.02)	0.129
Model 2	1	1.02 (0.85–1.22)	1.12 (0.93–1.34)	0.83 (0.68–1.01)	0.99 (0.97–1.02)	0.013
Model 3	1	1.04 (0.86–1.25)	1.12 (0.93–1.34)	0.84 (0.69–1.03)	1.00 (0.97–1.02)	0.048
High blood pressure						
Model 1	1	0.96 (0.79–1.15)	1.25 (1.03–1.52)	0.96 (0.79–1.17)	1.01 (0.98–1.03)	0.932
Model 2	1	0.95 (0.78–1.15)	1.24 (1.02–1.51)	0.98 (0.80–1.20)	1.01 (0.98–1.03)	0.898
Model 3	1	0.96 (0.79–1.17)	1.22 (0.99–1.49)	1.01 (0.82–1.24)	1.01 (0.98–1.04)	0.832
High blood glucose						
Model 1	1	0.73 (0.61–0.88)	0.67 (0.56–0.79)	0.54 (0.45–0.64)	0.93 (0.91–0.96)	< 0.001
Model 2	1	0.71 (0.59–0.85)	0.65 (0.54–0.77)	0.53 (0.44–0.64)	0.93 (0.91–0.96)	< 0.001
Model 3	1	0.72 (0.60–0.87)	0.63 (0.52–0.76)	0.53 (0.44–0.64)	0.93 (0.91–0.96)	< 0.001

Model 1: adjusted for age and sex;

Model 2: additionally adjusted for education, current smoking status, current alcohol drinking status, physical activity and family history of diabetes;

Model 3: additionally adjusted for BMI

Abbreviations: *MetS* Metabolic syndrome, *SD* Standard deviation, *HDL* High-density lipoprotein

In stratified analysis (Table 5), ORs (95% CIs) of the highest quartiles of all subgroups decreased significantly, indicating the robust association. No interaction was recognized between age, sex, BMI, physical activity, smoking, drinking alcohol and chromium (*P* for interaction > 0.05).

**Table 5:** Odds ratios for MetS of plasma chromium concentrations by subgroup

Participant	%	Quartiles of plasma chromium concentrations				Per 1 μg/L Chromium	*P* for interaction
		Q1	Q2	Q3	Q4		
subgroup							
Age							0.056
< 50	40.1	1	0.89 (0.69–1.16)	1.05 (0.81–1.37)	0.72 (0.52–0.98)	0.97 (0.91–1.03)	
≥ 50	59.9	1	0.76 (0.61–0.95)	0.66 (0.53–0.82)	0.60 (0.48–0.74)	0.92 (0.89–0.96)	
Sex							0.161
Women	39.6	1	0.87 (0.67–1.14)	0.98 (0.75–1.28)	0.76 (0.58–0.99)	0.96 (0.92–1.01)	
Men	60.4	1	0.73 (0.58–0.91)	0.69 (0.55–0.86)	0.56 (0.44–0.70)	0.91 (0.88–0.95)	
BMI							0.537
< 24	49.3	1	0.73 (0.56–0.95)	0.79 (0.60–1.03)	0.61 (0.46–0.80)	0.93 (0.89–0.98)	
≥ 24	50.7	1	0.93 (0.71–1.22)	0.78 (0.60–1.00)	0.68 (0.52–0.89)	0.94 (0.90–0.98)	
Physical activity							0.271
No	59.8	1	0.74 (0.60–0.93)	0.82 (0.66–1.03)	0.64 (0.51–0.81)	0.93 (0.89–0.97)	
Yes	40.2	1	0.89 (0.67–1.17)	0.76 (0.58–0.99)	0.62 (0.47–0.81)	0.94 (0.89–0.98)	
Smoking							0.229
No	69.2	1	0.85 (0.69–1.04)	0.86 (0.70–1.05)	0.70 (0.57–0.87)	0.94 (0.91–0.98)	
Yes	30.8	1	0.67 (0.49–0.92)	0.67 (0.49–0.91)	0.49 (0.35–0.67)	0.91 (0.86–0.97)	
Drinking alcohol							0.314
No	71.2	1	0.85 (0.70–1.04)	0.86 (0.70–1.05)	0.62 (0.50–0.76)	0.93 (0.90–0.96)	
Yes	28.8	1	0.65 (0.48–0.90)	0.67 (0.49–0.91)	0.67 (0.48–0.93)	0.94 (0.88–1.00)	

Adjusted for age, sex, BMI, education, current smoking status, current alcohol drinking status, physical activity and family history of diabetes

Abbreviations: *MetS* Metabolic syndrome, *SD* Standard deviation, *BMI* Body mass index

## Discussion

In this matched case-control study, we found that plasma chromium concentrations were inversely associated with the prevalence of MetS among Chinese adults. The inverse association was mainly explained by the relations between plasma chromium concentrations and waist circumference, the triglycerides and blood glucose levels. The associations were not appreciably changed by multivariate adjustment, and were consistent in the stratified analyses.

Chromium coming from foods varies and is usually very low [21]. Dietary intake of chromium from Asian diets ranged from 59.9 to 224 μg per day [22]. It is difficult in estimating dietary chromium due to its wide variability and low content in food sources, so a sensitive and reliable biomarker for chromium intake is required in epidemiological studies. Plasma chromium is considered a reliable objective biomarker for chromium exposure [23]. Previous studies reporting plasma chromium concentrations in large populations were sparse. Currently, there is no international acceptable value or range for the plasma chromium concentration in the general population. The mean concentration of plasma chromium in our population was 4.51 ± 2.24 μg/L, higher than the previously published studies, which varied from 0.2 to 0.86 μg/L [24–26]. A possible explanation for it may be higher contamination for the population. As some studies indicated that chromium exposure may come from industrial pollution like coal and oil combustion, the metal fabrication industry and the leather tanning sector, and China had a dramatic increase of anthropogenic chromium emissions from 1990 to 2009 [27].

There existed few high-quality evidence focused on the relationship between chromium and MetS at present. Limited epidemiological study yielded controversial results. A 23-year follow-up study including 3648 American adults indicated that toenail chromium levels were inversely and longitudinally associated with incidence of MetS [10]. However, another cross-sectional study conducted in Korea did not support the association between toenail chromium concentrations and MetS and its components [13].

In our study, significant associations between chromium concentrations and waist circumference, triglycerides and blood glucose levels were noticed. These associations might explain the latent mechanism involved in the relationship of chromium and MetS.

The association of plasma chromium with high blood glucose was the strongest among the components of MetS in this study. Although the pathogenesis of MetS remains unclear, recent interest has focused on the possible involvement of insulin resistance as a linking factor [28]. Coincident with this, our previous study has elaborated the inverse association between plasma chromium concentrations and type 2 diabetes mellitus and pre-diabetes mellitus in a case-control study [12]. In addition, evidences in animal and in vitro studies supported the association as well. A lot of studies demonstrated that chromium may up-regulate insulin-stimulated insulin signal transduction by a variety of mechanisms [5, 6, 8, 29–31]. However, it is worth concerning the causality of chromium status and high blood glucose. On one hand, the low levels of chromium might result in the diminution of insulin signal transduction, and further aggravate the development of insulin resistance. On the other hand, chromium lost and excreted from human body increased with aging and was related to the diabetes [32]. Large losses of chromium over more than 2 years’ diabetes duration may change the chromium homeostasis [33]. Further studies are warranted to investigate the causality of chromium status and high blood glucose.

Moreover, the effects of chromium on obesity and dyslipidemia has also been studied. The animal studies indicated that chromium might reduce the weight of obese rats and lipids levels as well [5, 9, 34, 35]. However, clinical trials were inconclusive with regard to weight control and lipid metabolism improvement. Although some studies claimed beneficial effects of chromium supplementation [36, 37], systematic reviews found it inadequate to inform firm decisions about the efficacy of chromium supplements on weight loss or lipid metabolism in overweight or obese adults because of the low-quality evidence [15, 18, 38].

The strengths of our study included the matched case-control study design, the large number of participants and objectively measured plasma chromium levels. In addition, chromium levels in plasma were measured using the state-of-the-art ICP-MS method. A few limitations need to be considered. First, the case-control nature of our study does not allow us to infer any causality and address temporal relationship between plasma chromium and MetS. Second, we could not differentiate trivalent chromium from hexavalent chromium in plasma measurement. Trivalent chromium is suggested to be beneficial and hexavalent chromium is toxic to human health [39]. Thus, the combination of these two forms may attenuate the association that may exist between trivalent chromium and MetS. Third, the classification of current smoking and alcohol drinking status and physical activity was not detailed enough. Additionally, the lack of information on the other unknown or unmeasured factors might also confound our results. Finally, the generalizability of our findings may be limited since all participants were of Chinese Han ethnicity. However, a homogenous ethnic background may reduce residual confounding from unmeasured genetic and cultural variability.

## Conclusions

Our study demonstrated an inverse association between plasma chromium levels and MetS in a Chinese population. The association was mainly accounted for the relations between plasma chromium levels and high waist circumference, and the triglycerides and blood glucose levels. Further studies are warranted to confirm our findings in prospective cohorts and to elucidate the potential mechanisms underlying the relationship between chromium and MetS, as well as MetS components.

## Supplementary information

**Additional file 1: Table S1** STROBE-nut: An extension of the STROBE statement for nutritional epidemiology.

**Additional file 2: Table S2** The Strengthening the Reporting Observational studies in Epidemiology – Molecular Epidemiology (STROBE-ME) Reporting Recommendations: Extended from STROBE statement.

## Data Availability

The datasets used and/or analysed during the current study are available from the corresponding author on reasonable request.

## Abbreviations

CI: Confidence interval
DBP: Diastolic blood pressure
MDA: Malonaldehyde
HDL: High-density lipoprotein
HOMA-β: Homeostasis model assessment of β-cell function
HOMA-IR: Homeostasis model assessment of insulin resistance
LOD: Limit of detection
LDL: Low-density lipoprotein
MetS: Metabolic syndrome
OR: Odds ratio
SBP: Systolic blood pressure
SD: Standard deviation
